# Characterization of Volatile Organic Compounds Emitted from Endophytic *Burkholderia cenocepacia* ETR-B22 by SPME-GC-MS and Their Inhibitory Activity against Various Plant Fungal Pathogens

**DOI:** 10.3390/molecules25173765

**Published:** 2020-08-19

**Authors:** Jian-Hua Chen, Wei Xiang, Ke-Xin Cao, Xuan Lu, Shao-Chang Yao, Ding Hung, Rong-Shao Huang, Liang-Bo Li

**Affiliations:** 1 College of Agriculture, Guangxi University, Nanning 530004, Guangxi, China; CJH1000@st.gxu.edu.cn (J.-H.C.); victorxiang@st.gxu.edu.cn (W.X.); 18991697639@163.com (K.-X.C.); 2 College of Pharmacy, Guangxi University of Chinese Medicine, Nanning 530200, Guangxi, China; lux@gxtcmu.edu.cn (X.L.); yaoshaochang@163.com (S.-C.Y.); dinghuang@gxtcmu.edu.cn (D.H.)

**Keywords:** volatile organic compounds, antifungal activity, biocontrol agents, bacteria, *Burkholderia cenocepacia*

## Abstract

The use of antagonistic microorganisms and their volatile organic compounds (VOCs) to control plant fungal pathogens is an eco-friendly and promising substitute for chemical fungicides. In this work, endophytic bacterium ETR-B22, isolated from the root of *Sophora tonkinensis* Gagnep., was found to exhibit strong antagonistic activity against 12 fungal pathogens found in agriculture. Strain ETR-B22 was identified as *Burkholderia cenocepacia* based on 16S rRNA and *recA* sequences. We evaluated the antifungal activity of VOCs emitted by ETR-B22. The VOCs from strain ETR-B22 also showed broad-spectrum antifungal activity against 12 fungal pathogens. The composition of the volatile profiles was analyzed based on headspace solid phase microextraction (HS-SPME) gas chromatography coupled to mass spectrometry (GC-MS). Different extraction strategies for the SPME process significantly affected the extraction efficiency of the VOCs. Thirty-two different VOCs were identified. Among the VOC of ETR-B22, dimethyl trisulfide, indole, methyl anthranilate, methyl salicylate, methyl benzoate, benzyl propionate, benzyl acetate, 3,5-di-tert-butylphenol, allyl benzyl ether and nonanoic acid showed broad-spectrum antifungal activity, and are key inhibitory compounds produced by strain ETR-B22 against various fungal pathogens. Our results suggest that the endophytic strain ETR-B22 and its VOCs have high potential for use as biological controls of plant fungal pathogens.

## 1. Introduction

Plant fungal pathogens cause diverse crop diseases resulting in drastic economic losses worldwide [1]. Improving the control of plant fungal diseases is an effective method for solving food shortage issues around the world [2,3]. Traditionally, chemical fungicides have been used to control fungal pathogens. However, intensive use of synthetic fungicides can cause problems such as pathogen resistance, fungicide residues, and environmental pollution [4,5,6]. There is an urgent need for environmentally friendly and effective agents to satisfy stricter agricultural product safety standards and increasing public concern.

The use of biocontrol microorganisms has attracted attention as a potential alternative to chemical measures in the control of plant pathogenic fungi [7]. Many antagonistic microorganisms, e.g., *Bacillus* [8], *Pseudomonas* [9], and *Burkholderia* [10], exhibit biocontrol activity that can control the growth of plant fungal pathogens. The antagonism of fungal pathogens by microorganisms mainly depends on their secretion of antimicrobial active compounds [11], competition for nutrients and living space [12], hyperparasitism and induction of systemic resistance responses in the host [13], and secretion of cell wall degrading enzymes (CWDEs) [14]. Additionally, volatile organic compounds (VOCs) released by microorganisms receive attention due to their antimicrobial effects [15,16]. Recently, VOCs produced by microorganisms were proposed as an alternative control method for fungal plant diseases because they can influence pathogenic fungal growth, deforming fungal hyphae and spores [17]. For instance, several VOCs (e.g., Pyrazine (2,5-dimethyl), benzothiazole, 4-chloro-3-methyl, and phenol-2,4-bis (1,1-dimethylethyl)) produced by *Bacillus velezensis* ZSY-1 exhibit significant antifungal activity against *Alternaria solani* and *Botrytis cinerea* [18]. On the other hand, VOCs can promote growth and induce systemic resistance in plants [19,20]. Therefore, it is suggested that VOCs from microorganisms are an important source for screening antimicrobial compounds and developing biological control agents (BCAs).

Endophytes, which colonize and survive in plants without damaging hosts or causing disease symptoms, are a better potential BCA resource [21]. Investigations show that endophytes also have different kinds of ecological functions, such as biocontrol activity [22], promotion of plant growth [23], and modulation of plant systemic resistance [24]. However, the VOCs emitted by endophytes have been overlooked due to the unique endophyte habitat. Therefore, increasing research effort in antimicrobial VOCs produced by endophytes seems particularly relevant.

*Sophora tonkinensis* Gagnep. is widely used as a traditional medicine and plant botanical fungicide source [25,26]. In a previous study, our laboratory screened and named the endophytic bacterium strain ETR-B22 isolated from the root of *S. tonkinensis*, which exhibited significant antifungal activity. However, its mechanism of antifungal action has not been fully elucidated. Therefore, the objective of this study was (1) to evaluate the antagonistic effects of stain ETR-B22; (2) to assess broad-spectrum antifungal effects of VOCs produced by strainETR-B22; (3) to identify and characterize the composition of the VOCs; and (4) to determine the chemical factors in pathogen inhibition.

## 2. Results

### 2.1. Molecular Identification and Phylogenetic Analysis

**Figure 1:**
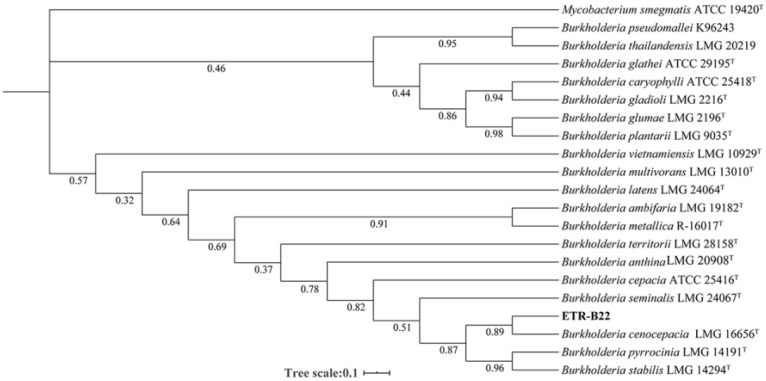
The Bayesian phylogenetic tree from the combined sequences of the16S rRNA and *recA* gene of strain ETR-B22 and type strains of the *Burkholderia* group. The tree is rooted with sequences of *Mycobacterium smegmatis* ATCC 19420. BI posterior probability (PP) is shown below the branch around the corresponding node. Strain ETR-B22 is highlighted in bold.

Endophytic bacterium ETR-B22 was identified using sequences of 16S rRNA and RecombinaseA (*recA*) genes. The lengths of the 16S rRNA and *recA* sequence of ETR-B22 described here are 1370 bp and 808 bp, respectively. Sequences of the 16S rRNA of ETR-B22 (accession number: MT712217) were blasted using the NCBI database, and the results show that they were identical to sequences of *B. cenocepacia* (LC462132) and *B. cepacia* (LC462132). Sequences of *recA* were used to further identify the species. Blast analysis of the *recA* gene showed that ETR-B22 (accession number: MT708133) was 98.89% similar to *B. cenocepacia* (AY951895). The phylogenetic tree indicates that strain ETR-B22 is sister to *B. cenocepacia* LMG 16,656 with a strong level of support (PP = 0.89) (Figure 1). This result showed that ETR-B22 was phylogenetically related to *B. cenocepacia* LMG 16,656 and that ETR-B22 was identified as *B. cenocepacia*.

### 2.2. In Vitro Antagonistic Assays of ETR-B22 against Pathogenic Fungus

**Figure 2:**
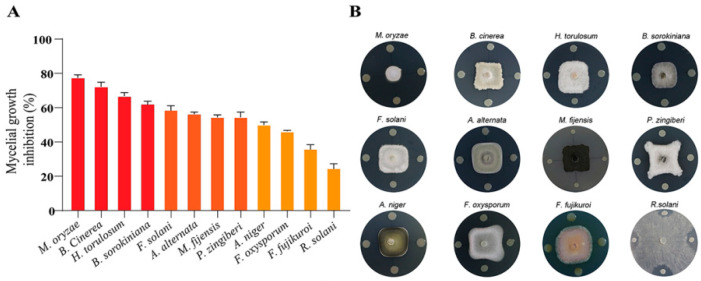
In vitro antagonism of strain ETR-B22 against 12 plant fungal pathogens using dual culture method. (**A**) Percentage of inhibition of fungal pathogens. Values represent the average of three replicates ± standard error. (**B**) Antagonism test between strain ETR-B22 and fungal pathogens.

The effect of dual plate tests indicated that ETR-B22 inhibited the mycelial growth of all tested pathogens (Figure 2B). The inhibition rates against most of the tested fungal pathogens were concentrated in the range between 24.0% and 77.8% (Figure 2A). Among the tested pathogens, *M. oryzae* was the most sensitive to strain ETR-B22 with the highest growth inhibition rate. ETR-B22 also significantly inhibited the growth of *B. cinerea* and *H. torulosum* at 72.5% and 66.9%, respectively. However, strain ETR-B22 only weakly affected *F. fujikuroi* and *R. solani*, with inhibition rates of less than 40.0%.

### 2.3. Antifungal Effect of VOCs Produced by ETR-B22

**Figure 3:**
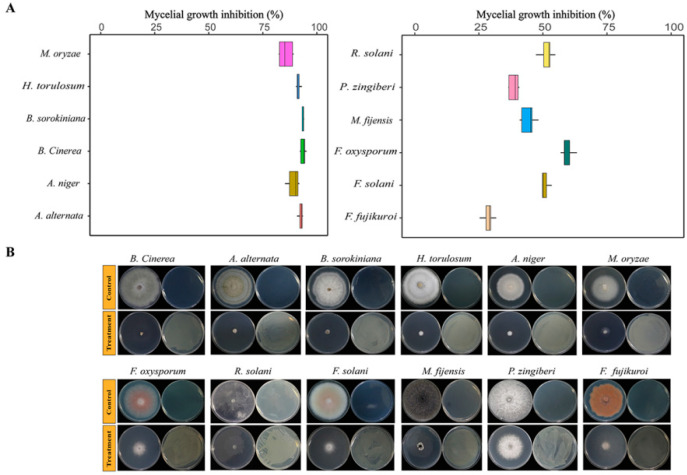
Antifungal effects of the volatile organic compounds produced by ETR-B22. (**A**) Box plots showed the inhibition rates of fungal growth exposure to VOCs produced by ETR-B22. Boxes represented the mean with standard errors of six independent biological replicates. (**B**) Mycelial growth inhibition of fungal pathogens by VOCs produced by ETR-B22.

In this study, VOCs produced by strain ETR-B22 significantly inhibited the mycelial growth of all tested pathogens. The inhibition of all tested pathogens showed slow colony growth and abnormal culture traits (Figure 3B). The pigment produced by Fusarium sp. or H. torulosum was significantly reduced after fumigation with VOCs. The effect of the VOCs produced by ETR-B22 on mycelial growth in pathogens was shown in Figure 3A. In our tests, different fungal strains exhibited different levels of sensitivity to VOCs produced by ETR-B22, with significant inhibition by 30.7%–93.7% (Figure 3A). The inhibition rate exceeded 90% suppression of four pathogens, namely, B. cinerea (94.6%), A. alternate (93.2%), B. sorokiniana (92.7%), and H. torulosum (92.0%).

### 2.4. Analysis of the VOCs Produced by ETR-B22

#### 2.4.1. Optimization of Extraction Temperature and Time of VOCs

**Figure 4:**
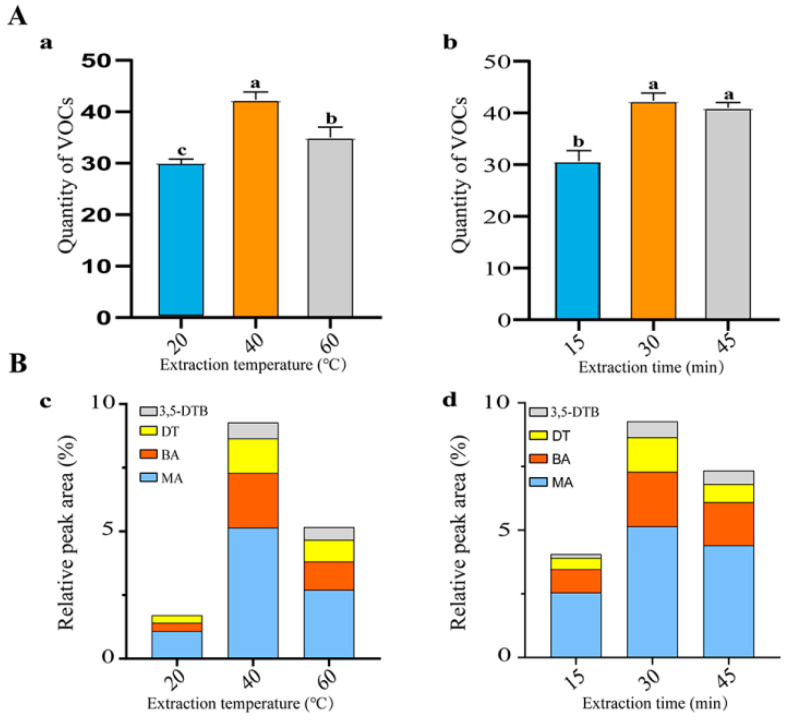
Effect of different extraction conditions on the GC–MS determination. (**A**) Quantity of VOCs statistics. (**a**) Different extraction temperatures. Values represented the average of three replicates ± standard error. Means with different letters indicate the statistical significance at *p* < 0.05. (**b**) Different extraction times. (**B**) Relative peak area of main compounds. (**c**) Different extraction temperatures. (**d**) Different extraction times. The main compounds: dimethyl trisulfide (DT); methyl anthranilate (MA); benzyl acetate (BA); 3,5-di-tert-butylphenol (3,5-DTB).

The results obtained from GC-MS showed that the characterization of VOCs was significantly affected by temperature and time of HS-SPME. In this case, the highest number of VOC compounds and highest relative abundance of representative compounds were confirmed when the extraction temperature was 40 °C at the same extraction time (30 min). Specifically, 42 distinct VOCs were identified at an extraction temperature of 40 °C, while only 31 and 35 distinct VOCs were resolved at 20 °C and 60 °C, respectively, when tested at the same extraction time (30 min) (Figure 4a). The GC peak relative area (RA) of representative compounds was greater at 40 °C than at 20 or 60 °C (Figure 4c). Moreover, 3,5-di-tert-butylphenol could not be detected when the extraction temperature was 20 °C. Thus, 40 °C was considered the best extraction temperature. GC results were also significantly influenced by the extraction time. When the extraction times were 15, 30, and 45 min, GC–MS resolved 33, 42, and 40 distinct VOCs, respectively (Figure 4b). The RA of the four main compounds was highest at an extraction time of 30 min (Figure 4d).

#### 2.4.2. Chemical Composition of VOCs Produced by ETR-B22 Analyzed by GC-MS

The chemical profiles of identified VOCs detected by GC-MS are listed in Table S1. A total 32 distinct VOCs were detected from the VOCs of ETR-B22. The major identified compounds of ETR-B22 included the following nine structurally distinct classes: ethers, esters, ketones, aldehydes, alcohols, hydrocarbons, phenols, organic acids and nitrogenous compounds. Esters and hydrocarbons were the main compound types, accounting for 59.4% of the 32 detected compounds. Based on the relative peak area, the compound methyl anthranilate was the most abundant volatile, followed by benzyl acetate. The VOCs identified by GC-MS were chosen as target VOCs in the following experiment.

### 2.5. In Vitro Antagonistic Activity of Selected Individual VOCs

Fourteen pure standards were tested individually against 12 fungal pathogens. As shown in Table 1, some compounds showed antifungal activity against some or all tested pathogens. Among the 14 compounds tests, compound 1 and 8 showed antifungal activities indicating that the growth of all pathogens was completely inhibited (100% suppression). Additionally, compounds 3, 4, 5, 6, and 9 exhibited broad spectrum antifungal activity against all fungal pathogens. Compounds 2 and 7 exhibited limited antifungal activity, as not all pathogens were sensitive to these. Compounds 11, 12, 13, and14 showed poor antifungal activity or no mycelial growth against all pathogens in our test.

## 3. Discussion

Although the screening of a BCA’s sources mainly focuses on the rhizosphere and rhizoplane soil, there has been increasing interest over the last few years in using bacterial endophytes as biological control agents [27]. The endophytic strain ETR-B22 isolated from *S. tonkinensis* was identified as *B. cenocepacia*, which belongs to *B. cepacia* complex (BCC) [28]. Bcc occupy a surprisingly wide range of ecological niches and have useful biotechnological potential associated with biological control, plant growth promotion and degradative agents of aromatic pollutants [29,30,31]. Here, we focused on the broad spectrum of ETR-B22 against plant pathogenic fungi, including nine genera and twelve species. Previous research on biological control agents based on *B. cenocepacia* has been reported in several plant diseases and reached the stage of commercialization [32]. However, the antifungal activity of *B. cenocepacia* against *Magnaporthe oryzae*, *Aspergillus niger*, *Bipolaris sorokiniana*, *Helminthosporium torulosum*, *Mycosphaerella fijensis* and *Phyllosticta zingiberi* has not been reported. Specifically, ETR-B22 exhibited significant antagonistic activity against *M. oryzae* (77.5% suppression), which is a destructive pathogen that threatens cultivated rice. Thus, compared with other *B. cenocepacia* strains used against plant fungal pathogens that have been reported [33,34], ETR-B22 exhibits a stronger antifungal effect against a broader spectrum of fungal pathogens. Our study further highlights the potential role of *B. cenocepacia* ERT-B22 as a biological agent for controlling plant pathogenic fungi.

The volatile organic compounds produced by biocontrol bacteria attract wide attention for their antifungal effects against several important fungal phytopathogens. Compared with non-volatile antifungal substances, bacterial VOCs have some significant benefits: they have smaller molecules, longer-distance range of action, and degrade easily [35]. In our study, we found that the VOCs produced by ETR-B22 showed strong antagonistic activity against a variety of fungal pathogens. Although ETR-B22 had a wide-spectrum antifungal activity against all of the tested fungal pathogens, genus–genus or species–species differences were observed. For instance, compared to *Fusarium* species, some genera were more sensitive. Similarly, within the same genus, some species were more sensitive than others. Comparing the inhibition rate of the VOCs against the three *Fusarium* species, it was found that the inhibition rate against *F. solani* was higher than that of *F. fujikuroi*. This result may be explained by antifungal mechanisms or chemical differences among taxa. For *Burkholderia* spp., similar results have been reported by other authors. For instance, the VOCs produced by a *B. gladioli* strain exert antifungal activity against plant pathogenic fungi by producing diffusible metabolites [36]. The VOCs produced by *B. tropica* inhibited the growth of fungal pathogens mycelium and disrupted fungal hyphae [37]. To the best of our knowledge, our study was the first to report that the antifungal and biological control potential of VOCs produced by endophytic *B. cenocepacia* that significant growth inhibition (i.e., 75%+ suppression rate) was found for six fungi, while three were below 50% growth inhibition. Our results suggest that the VOCs produced by ETR-B22 represent an effective biocontrol tool to protect plants against pathogen infection.

Solid phase micro-extraction technology was used to extract VOCs. The extraction temperature and time are the main factors that determine the efficiency of extraction. In this study, 40 °C for 30 min was considered the optimal condition to extract VOCs from ETR-B22 strain (Figure 4). Even though there have been some studies about different extraction temperature and time of VOCs produced by Burkholderiaceae, little is known about the optimal combination. A work conducted by Hazem previously handled volatiles emitted by *B. gladioli* with extraction conditions at 20 °C for 30 min [36]. However, we notice that this extraction condition is not appropriate for *B.cenocapacia* ETR-B22, since the number of detected volatiles significantly decreased. Furthermore, microorganisms with various ecologies, habitats and genotypes possess different sensitivity to temperature, causing the complex and diverse impact of VOCs extraction temperature [38,39,40]. It has long been known that molecular motion and volatility of volatiles depend on temperature. Compound 3,5-DTB may have lower molecular motion and volatility at 20 °C, so it was not detected at this temperature (Figure 4d). Extraction time is related to the saturability of adsorption capacity of the extraction column [41]. Therefore, the extraction column adsorbed volatiles incompletely in a short time (20 min), while the adsorbed compounds were degraded in a long time (40 min). Additionally, variations to other conditions, such as extraction fibers and GC conditions, can be further researched [42].

On the basis of GC−MS analysis, esters and hydrocarbons were the main compound types of VOCs emitted by strain ETR-B22. Some volatiles produced by Burkholderiaceae have been previously identified. For instance, 1-methyl-4-(1-methylethenyl)-cyclohexene is one of the main compounds of VOCs produced by *B. gladioli* strain ICMP 11,096 [36]; and sulfur compounds, ketones, and aromatic compounds were major groups in volatile profiles produced by *B. ambifaria* [43]. However, the characteristics and main compositions of volatiles produced by strain ETR-B22 are distinct from other *Burkholderia* species. This indicated that the taxonomic status of microorganisms has a greater influence on the chemical composition of VOCs. Additionally, there is a lack of research on volatile profiles produced by *B. cenocepacia*. This work therefore contributes to existing knowledge of volatile composition produced by *B. cenocepacia* by identifying the VOCs emitted from ETR-B22.

By screening in vitro, some known VOCs with antifungal effects were detected in our analyses, including dimethyl trisulfide [44], indole [45], methyl anthranilate [46], methyl salicylate [47], benzyl acetate [48], 3,5-di-tert-butylphenol [49], and nonanoic acid [50]. Nevertheless, these volatile compounds have not been reported in the VOCs produced by *Burkholderia* species except for dimethyl trisulfide. This work investigates, for the first time, the antifungal capability of allyl benzyl ether emitted from bacteria, which has potential as biological fungicide. A total of ten individual volatiles with antifungal activities are considered the key chemical composition of antifungal VOCs produced by strain ETR-B22. We noted that ETR-B22 significantly inhibited the growth of various fungi, which may have been due to one, some, or all of these bioactive VOCs. It has been reported previously that microbial VOCs are produced in combinations, the sum of which are responsible for antifungal activity [51,52]. Although we have tried to identify individual antifungal compounds, it should be noted that a certain set of VOCs is likely to be responsible for the growth inhibition effects we observed. Further research would be needed on investigating the degree to which the nine antifungal VOCs can inhibit growth independently, in combination, or in complex with the many other VOCs produced by ETR-B22. In addition, the concentration of VOCs has a potent influence on inhibitory effect [53,54]. The production and concentration of microbial VOCs necessitates further evaluation to confirm the efficacy of the biological control. Overall, our data represent a preliminary foundation for the clarification of key biocontrol VOCs produced by ETR-B22.

## 4. Conclusions

This work confirmed the broad-spectrum antifungal activity of *B. cenocepacia* strain ETR-B22. We investigated the potential of the volatile compounds as an effective mechanism of action against a wide range of crop fungal pathogens. Based on HS-SPME-GC-MS, extraction temperature and time of VOCs were optimized, and thirty-two probable VOCs were characterized and identified. The ten individual volatiles with antifungal activities, including dimethyl trisulfide, indole, methyl anthranilate, methyl salicylate, methyl benzoate, benzyl propionate, benzyl acetate, 3,5-di-tert-butylphenol, allyl benzyl ether and nonanoic acid, were key inhibitory VOCs which were promising candidates for bio-fungicide development. This study reports for the first time the characterization of VOCs produced by endophytic *B. cenocepacia* ETR-B22 and their individually inhibitory effects. Therefore, the results are useful in informing more applied research on *B. cenocepacia* as BAC against fungal pathogens. Further studies need to focus on designing agriculturally acceptable and practical ways for efficient use of *B. cenocepacia* ETR-B22 and its antifungal volatiles to control crop fungal disease.

## 5. Materials and Methods

### 5.1. Endophytic Bacterium and Plant Fungal Pathogens

Endophytic bacterium ETR-B22 was isolated from wide *S. tonkinensis* roots from the locality of traditional geo-authentic production areas in Guangxi province of south China. Strain ETR-B22 with antifungal activity was selected for further experiments. For culture condition, ETR-B22 was grown in Nutrient Agar (NA) medium with pH 7.4 at 30 °C. The bacterial suspension in 25% glycerol was preserved at −80 °C and frozen for long-term storage.

The fungal pathogens used in the experiment were *Alternaria alternata*, *Aspergillus niger*, *Bipolaris sorokiniana,* *Botrytis cinerea*, *Fusarium solani*, *F. oxysporum*, *F. fujikuroi*, *Helminthosporium torulosum*, *Mycosphaerella fijensis*, *Magnaporthe oryzae,* *Phyllosticta zingiberi* and *Rhizoctonia solani*. The above strains were provided by the Institute of Plant Pathology, College of Agriculture, Guangxi University, Guangxi, China, and stored in our laboratory. They were cultivated on potato dextrose agar (PDA) at 28 °C for 10–15 days and maintained at 4 °C for up to 2 months prior to use. For long-term storage, they were preserved with spores or mycelia in 25% (*v*/*v*) glycerol at −80 °C.

### 5.2. Molecular Identification and Phylogenetic Analysis

The DNA of strain ETR-B22 was extracted using FastPure Bacteria DNA Isolation Mini Kit (Vazyme Biotech Co., Ltd., Nanjing, China) following the manufacturer’s instructions. The universal primers 27F (5′-CAGAGTTTGATCCTGGCT-3′) and 1492R (5′-AGGAGGTGATCCAGCCGCA-3′) were used to amplify and sequence the 16s rRNA gene. Based on 16S rRNA sequencing, ETR-B22 was initially identified as *Burkholderia* sp. To identify it at the species level, the primers BUR1 (5′-GATCGARAAGCAGTTCGGCAA-3′) and BUR2 (5′-TTGTCCTTGCCCTGRCCGAT-3′) were used to sequence the housekeeping gene *recA* [55]. PCR products were sequenced at Sangon Biotech in Shanghai, China. The obtained sequences were analyzed to identify the strain with the BLAST searches in public databases (NCBI, USA). Type strains of the *Burkholderia* group were collected to construct a phylogenetic tree and the taxonomic status of strain ETR-B22 was analyzed. Briefly, partial sequences of *recA* (518 bp) and 16S rRNA (1334 bp) genes of the *Burkholderia* strain were concatenated to yield an alignment of 1852 sites. A concatenated phylogenetic tree supplemented with sequences of the *Burkholderia* group was constructed using Bayesian inference (BI) analysis with optimal substitution model GTR+G+I selected by MrModelTest 2.4 according to the Akaike Information Criterion (AIC). All BI analyses were run for 2,000,000 generations with four chains in two parallel runs and sampled every 5000 generations with a burn-in of the first 5000 trees. The convergence of the two parallel runs was determined by a splitting frequency of less than 0.005. All other parameters were set as default. Sequences of the 16S rRNA (accession numbers: MT712217) and *recA* gene (accession numbers: MT708133) were deposited.

### 5.3. In Vitro Antagonistic Assays of ETR-B22 Against Pathogenic Fungus

The in vitro antagonistic activity of strain ETR-B22 against 12 pathogenic fungi was tested using the dual culture method [56]. A 4 mm diameter mycelial disc of each 10-day old pathogenic fungus was inoculated towards the center of the dish containing approximately 25 mL of PDA and 1 μL cell suspension of strain ETR-B22 (10^7^ CFU/mL) was pointed in a cross shape at a distance of 3 cm from the mycelial disc. Three replications were performed for each treatment. In addition, mycelial discs were placed on PDA plates without any ETR-B22 strain as a negative control. The cultures were incubated at 28 ± 1 °C in darkness for 12 days. The relative inhibition (RI) was then calculated with the formula:

RI (%) = [(radial growth in control − radial growth in samples)/radial growth in control] × 100.(1)

### 5.4. Antifungal Effect of VOCs Produced by ETR-B22

The antifungal activity of the VOCs emitted by strain ETR-B22 was evaluated against 12 fungal pathogens using the two sealed base plates assay according to the method of Kong et al. [57]. For this purpose, 10 μL of ETR-B22 cell suspension (10^7^ CFU/mL) was sprayed on one base plate containing NA media, and a 5 mm diameter mycelial disc of pathogenic fungi was placed on the other base plate containing PDA media. One plate with pathogenic fungi and the other with sterile water served as the control. These two base plates were sealed with a double layer of Parafilm and cultured at 28 °C. There were six replicates of all treatments and controls. The diameters of each fungi colony were measured by Vernier caliper when the fungal hyphae reached the edge of the control plates. The inhibition rate of mycelial growth was calculated on the basis of the difference between the treatment and control according to the formula:

(2) $$\mathrm{Inhibition}\;\mathrm{rate}\;\left(\%\right)=\left(1-\frac{\mathrm{Dt}-\mathrm{Dd}}{\mathrm{Dc}-\mathrm{Dd}}\right)\times 100\%$$

where Dc is the average diameter of the mycelial colony in the control plate, Dt is that of the test plate, and Dd is that of the inoculated mycelium discs.

### 5.5. Analysis of the VOCs Produced by ETR-B22

#### 5.5.1. Volatiles Extraction and Optimization of Extraction Conditions Using Headspace Solid Phase Microextraction

A sterile 25 mL headspace vial containing 5 mL NA medium was prepared. A total of 20 μL of ETR-B22 cell suspension (10^7^ CFU/mL) was inoculated on the surface of NA medium. An inoculation with 20 μL sterile water was used as the control. All cultures were incubated at 30 °C for 5 days. Headspace solid phase microextraction (HS-SPME) was used to extract the VOCs. An SPME fiber (2 cm- 50/30 μm DVB/CAR/PDMS, Supelco Inc, Bellefonte, PA, USA) was performed to adsorb VOCs. According to the recommendations of manufacturer, the fiber was preconditioned at 250 °C for 30 min to remove residual compounds before extraction. Trapped compounds were then thermally desorbed from the fiber for 2 min in the GC injection port at 250 °C in the split-less injection mode.

To enhance extraction efficiency, the extraction temperature and time were optimized. The extraction process was set to 30 min at 20 °C, 40 °C, and 60 °C. Then, the extraction time was optimized at the optimal temperature for 15 min, 30 min, and 45 min. All samples were then characterized using HS-SPME coupled with gas chromatography and mass spectrometry analysis. In this test, the total number of compounds and relative peak area of main compounds in different extraction conditions were compared in the chromatogram.

#### 5.5.2. Identification of VOCs by GC-MS

For peak separation and detection, a GC 2010 gas chromatograph equipped with a HP-5 fused silica capillary column (30 m × 0.25 mm × i.d. × 0.25 μm) connected to a triple quadrupole mass spectrometer (SHIMADZU GC-2010 Plus GC system and SHIMADZU TQ8040 mass spectrometer) was used. The chromatographic conditions were as follows: the injection port was heated at 250 °C. Helium was used as a carrier gas, and the flow rate was 1 mL/min. The GC temperature program was set at 50 °C for 2 min, after which it was programed to increase from 40 to 150 °C by 4 °C/min, maintained at 150 °C for 1 min, increased from 160 to 250 °C by 5 °C/min, and finally maintained at 250 °C for 5 min. The following conditions of MS were used: (i) The ion source was electrospray ionization (ESI); (ii) the transfer line and the ion source was heated at 250 °C; (iii) the mass spectrometer was operated in the electron impact of 70e V, scanning the range of 45/500 m/z in full scan acquisition mode, with a scan rate of 0.2 scan/s. The mass spectra and retention time of each GC peak in the total ion chromatography were compared with the data system library (NIST 11 MS Library). The relative abundance of each compound was estimated by relative area (RA). A blank experiment (growth medium not inoculated with ETR-B22) was performed under the same conditions. All measurements were made with three replicates.

### 5.6. In Vitro Antagonistic Activity of Selected Individual VOCs

Fourteen pure standards—produced by ETR-B22 and previously identified through GC-MS analysis—were selected to individually confirm their antifungal activity. All pure standards of VOCs were purchased from Sigma-Aldrich (St. Louis, MO, USA). For this purpose, an in vitro two sealed base plates assay was used. Mycelial agar discs with a diameter of 5 mm were placed in the center of petri dishes containing a PDA medium depending on the pathogens. A sterile paper filter with 50 μL of pure standard was placed in the center of another petri dish. Then, the petri dishes were double sealed with Parafilm and incubated for 10 days at 28 °C. As a control, pure standards were replaced by the equivalent amounts of distilled water. The sample unit was represented by three replicates for each standard and pathogen. The inhibition rate of fungal growth was calculated according to the formula described in Section 2.2.

### 5.7. Statistical Analysis

The final experimental data were expressed as mean and the standard deviation (SD), calculated using SPSS Statistics version 17.0. Differences among groups were compared using one-way analysis of variance (ANOVA) followed by Duncan’s multiple range test. The results were considered to be statistically significant at *p* < 0.05.

## Figures and Tables

**Table 1:** Mycelial growth of plant fungal pathogens exposed to 14 pure standards after 10 days.

**No.**	**Compound**	**Inhibition Rate (%)**					
		**Aa**	**An**	**Bc**	**Bs**	**Fs**	**Fo**
1	Dimethyl trisulfide	100 ± 0.00	100 ± 0.00	100 ± 0.00	100 ± 0.00	100 ± 0.00	100 ± 0.00
2	Allyl benzyl ether	66.87 ± 2.91	34.55 ± 4.14	86.71 ± 0.58	67.18 ± 0.59	-	-
3	Methyl benzoate	89.42 ± 1.45	67.15 ± 3.23	100 ± 0.00	84.26 ± 0.36	25.68 ± 0.82	67.77 ± 0.25
4	Benzyl acetate	75.76 ± 1.86	81.19 ± 2.47	91.28 ± 0.27	76.27 ± 0.52	22.57 ± 0.44	53.85 ± 2.03
5	Methyl salicylate	100 ± 0.00	76.23 ± 2.92	100 ± 0.00	87.19 ± 1.46	40.42 ± 4.27	100 ± 0.00
6	Benzyl propionate	77.91 ± 2.38	52.28 ± 1.14	92.23 ± 0.25	100 ± 0.00	32.26 ± 1.29	82.45 ± 1.14
7	Nonanoic acid	80.28 ± 1.94	47.18 ± 1.54	83.28 ± 1.89	48.03 ± 1.10	-	-
8	Indole	100 ± 0.00	100 ± 0.00	100 ± 0.00	100 ± 0.00	100 ± 0.00	100 ± 0.00
9	Methyl anthranilate	82.07 ± 2.47	76.56 ± 1.20	88.27 ± 0.72	79.24 ± 1.23	55.17 ± 1.14	70.25 ± 0.77
10	3,5-Di-tert-butylphenol	78.33 ± 4.05	71.61 ± 0.71	87.67 ± 1.39	75.84 ± 1.67	53.18 ± 0.73	68.09 ± 0.95
11	3-Hexen-1-ol, benzoate, (Z)-	7.90 ± 2.25	-	17.94 ± 1.29	-	-	-
12	2-Pentadecanone	-	-	-	16.36 ± 1.98	-	-
13	Dodecanamide	-	-	-	14.95 ± 1.24	-	-
14	Benzyl benzoate	-	24.62 ± 1.98	-	-	-	-
**No** **.**	**Compound**	**Inhibition Rate (%)**					
		**Ff**	**Ht**	**Mf**	**Mo**	**Pz**	**Rs**
1	Dimethyl trisulfide	100 ± 0.00	100 ± 0.00	100 ± 0.00	100 ± 0.00	100 ± 0.00	100 ± 0.00
2	Allyl benzyl ether	53.48 ± 0.79	25.86 ± 0.41	61.53 ± 2.21	36.65 ± 1.18	78.32 ± 0.51	56.28 ± 0.94
3	Methyl benzoate	70.36 ± 0.54	83.15 ± 1.29	11.37 ± 0.31	85.64 ± 2.58	100 ± 0.00	67.29 ± 0.68
4	Benzyl acetate	67.45 ± 0.86	65.16 ± 4.60	38.81 ± 1.63	91.29 ± 1.47	40.29 ± 0.63	74.68 ± 1.34
5	Methyl salicylate	82.72 ± 0.22	87.76 ± 1.38	100 ± 0.00	100 ± 0.00	100 ± 0.00	65.05 ± 1.41
6	Benzyl propionate	79.65 ± 1.87	73.38 ± 0.77	18.05 ± 0.92	79.59 ± 0.71	80.92 ± 1.27	36.21 ± 0.30
7	Nonanoic acid	78.85 ± 3.37	100 ± 0.00	-	52.38 ± 0.38	44.21 ± 1.05	62.02 ± 0.61
8	Indole	100 ± 0.00	100 ± 0.00	100 ± 0.00	100 ± 0.00	100 ± 0.00	100 ± 0.00
9	Methyl anthranilate	83.75 ± 0.31	90.54 ± 0.64	18.44 ± 0.37	100 ± 0.00	58.12 ± 0.74	84.87 ± 0.97
10	3,5-Di-tert-butylphenol	77.08 ± 0.14	77.65 ± 0.68	27.69 ± 1.19	81.35 ± 0.48	84.36 ± 1.75	82.31 ± 1.82
11	3-Hexen-1-ol, benzoate, (Z)-	-	-	-	9.76 ± 0.61	-	-
12	2-Pentadecanone	-	-	-	-	-	13.74 ± 0.27
13	Dodecanamide	-	-	-	-	-	-
14	Benzyl benzoate	-	-	-	-	-	10.52 ± 1.17

Note: “-” indicates no antifungal activity. Data were presented as means ± standard error. Twelve different fungal strains including *A. alternate* (Aa), *Aspergillus niger* (An), *B. sorokiniana* (Bs), *B. Cinerea* (Bc), *F. solani* (Fs), *F. oxysporum* (Fo), *F. fujikuroi* (Ff), *H. torulosum* (Ht), *M. fijensis* (Mf), *M. oryzae* (Mo), *P. zingiberi* (Pz) and *R. solani* (Rs) were used in the tests.

## Supplementary Materials

The Supplementary Materials are available online.

## References

[1] Bebber D.P., Gurr S.J. (2015). Crop-destroying fungal and oomycete pathogens challenge food security. Fungal Genet. Biol..

[2] Hossain M.I., Sadekuzzaman M., Ha S.-D. (2017). Probiotics as potential alternative biocontrol agents in the agriculture and food industries: A review. Food Res. Int..

[3] Raymaekers K., Ponet L., Holtappels D., Berckmans B., Cammue B.P.A. (2020). Screening for novel biocontrol agents applicable in plant disease management—A review. Biol. Control.

[4] Schreinemachers P., Tipraqsa P. (2012). Agricultural pesticides and land use intensification in high, middle and low income countries. Food Policy.

[5] Food and Agriculture Organization of the United Nations (2017). The Future of Food and Agriculture: Trends and Challenges.

[6] Zhang Y., Li T., Liu Y., Li X., Zhang C., Feng Z., Peng X., Li Z., Qin S., Xing K. (2019). Volatile Organic Compounds Produced by *Pseudomonas chlororaphis* subsp. *aureofaciens* SPS-41 as Biological Fumigants To Control *Ceratocystis fimbriata* in Postharvest Sweet Potatoes. J. Agric. Food Chem..

[7] Parnell J.J., Berka R., Young H.A., Sturino J.M., Kang Y., Barnhart D.M., DiLeo M.V. (2016). From the Lab to the Farm: An Industrial Perspective of Plant Beneficial Microorganisms. Front. Plant Sci..

[8] Villa-Rodríguez E., Parra-Cota F., Castro-Longoria E., López-Cervantes J., de los Santos-Villalobos S. (2019). Bacillus subtilis TE3: A promising biological control agent against Bipolaris sorokiniana, the causal agent of spot blotch in wheat (*Triticum turgidum* L. subsp. durum). Biol. Control.

[9] Mishra J., Arora N.K. (2018). Secondary metabolites of fluorescent pseudomonads in biocontrol of phytopathogens for sustainable agriculture. Appl. Soil Ecol..

[10] Jung B.K., Hong S.-J., Park G.-S., Kim M.-C., Shin J.-H. (2018). Isolation of Burkholderia cepacia JBK9 with plant growth-promoting activity while producing pyrrolnitrin antagonistic to plant fungal diseases. Appl. Biol. Chem..

[11] Raaijmakers J.M., Mazzola M. (2012). Diversity and Natural Functions of Antibiotics Produced by Beneficial and Plant Pathogenic Bacteria. Annu. Rev. Phytopathol..

[12] Bencheqroun S.K., Bajji M., Massart S., Labhilili M., Jaafari S.E., Jijakli M.H. (2007). In vitro and in situ study of postharvest apple blue mold biocontrol by Aureobasidium pullulans: Evidence for the involvement of competition for nutrients. Postharvest Biol. Technol..

[13] Wiesel L., Newton A.C., Elliott I., Booty D., Gilroy E.M., Birch P.R.J., Hein I. (2014). Molecular effects of resistance elicitors from biological origin and their potential for crop protection. Front. Plant Sci..

[14] Angeli D., Puopolo G., Maurhofer M., Gessler C., Pertot I. (2012). Is the mycoparasitic activity of Ampelomyces quisqualis biocontrol strains related to phylogeny and hydrolytic enzyme production?. Biol. Control.

[15] Raza W., Ling N., Yang L., Huang Q., Shen Q. (2016). Response of tomato wilt pathogen Ralstonia solanacearum to the volatile organic compounds produced by a biocontrol strain Bacillus amyloliquefaciens SQR-9. Sci. Rep..

[16] Alijani Z., Amini J., Ashengroph M., Bahramnejad B. (2019). Antifungal activity of volatile compounds produced by Staphylococcus sciuri strain MarR44 and its potential for the biocontrol of Colletotrichum nymphaeae, causal agent strawberry anthracnose. Int. J. Food Microbiol..

[17] Boukaew S., Petlamul W., Bunkrongcheap R., Chookaew T., Kabbua T., Thippated A., Prasertsan P. (2018). Fumigant activity of volatile compounds of Streptomyces philanthi RM-1-138 and pure chemicals (acetophenone and phenylethyl alcohol) against anthracnose pathogen in postharvest chili fruit. Crop Prot..

[18] Gao Z., Zhang B., Liu H., Han J., Zhang Y. (2017). Identification of endophytic Bacillus velezensis ZSY-1 strain and antifungal activity of its volatile compounds against Alternaria solani and Botrytis cinerea. Biol. Control.

[19] Ryu C.-M., Farag M.A., Hu C.-H., Reddy M.S., Kloepper J.W., Paré P.W. (2004). Bacterial Volatiles Induce Systemic Resistance in Arabidopsis. Plant Physiol..

[20] Farag M.A., Ryu C.-M., Sumner L.W., Paré P.W. (2006). GC–MS SPME profiling of rhizobacterial volatiles reveals prospective inducers of growth promotion and induced systemic resistance in plants. Phytochemistry.

[21] Eljounaidi K., Lee S.K., Bae H. (2016). Bacterial endophytes as potential biocontrol agents of vascular wilt diseases—Review and future prospects. Biol. Control.

[22] Wicaksono W.A., Jones E.E., Casonato S., Monk J., Ridgway H.J. (2018). Biological control of Pseudomonas syringae pv. actinidiae (Psa), the causal agent of bacterial canker of kiwifruit, using endophytic bacteria recovered from a medicinal plant. Biol. Control.

[23] Vurukonda S.S.K.P., Giovanardi D., Stefani E. (2018). Plant Growth Promoting and Biocontrol Activity of Streptomyces spp. as Endophytes. IJMS.

[24] Mishra A., Singh S.P., Mahfooz S., Singh S.P., Bhattacharya A., Mishra N., Nautiyal C.S. (2018). Endophyte-Mediated Modulation of Defense-Related Genes and Systemic Resistance in *Withania somnifera* (L.) Dunal under *Alternaria alternata* Stress. Appl. Environ. Microbiol..

[25] Zanardi O.Z., Ribeiro L.d.P., Ansante T.F., Santos M.S., Bordini G.P., Yamamoto P.T., Vendramim J.D. (2015). Bioactivity of a matrine-based biopesticide against four pest species of agricultural importance. Crop Prot..

[26] He L.-J., Liu J.-S., Luo D., Zheng Y.-R., Zhang Y.-B., Wang G.-C., Li Y.-L. (2019). Quinolizidine alkaloids from Sophora tonkinensis and their anti-inflammatory activities. Fitoterapia.

[27] Compant S., Brader G., Muzammil S., Sessitsch A., Lebrihi A., Mathieu F. (2013). Use of beneficial bacteria and their secondary metabolites to control grapevine pathogen diseases. BioControl.

[28] Coenye T., Vandamme P. (2003). Diversity and significance of Burkholderia species occupying diverse ecological niches. Environ. Microbiol..

[29] You M., Fang S., MacDonald J., Xu J., Yuan Z.-C. (2020). Isolation and characterization of Burkholderia cenocepacia CR318, a phosphate solubilizing bacterium promoting corn growth. Microbiol. Res..

[30] Morya R., Salvachúa D., Thakur I.S. (2020). Burkholderia: An Untapped but Promising Bacterial Genus for the Conversion of Aromatic Compounds. Trends Biotechnol..

[31] Ho Y.-N., Chiang H.-M., Chao C.-P., Su C.-C., Hsu H.-F., Guo C., Hsieh J.-L., Huang C.-C. (2015). In planta biocontrol of soilborne Fusarium wilt of banana through a plant endophytic bacterium, Burkholderia cenocepacia 869T2. Plant Soil.

[32] Chiarini L., Bevivino A., Dalmastri C., Tabacchioni S., Visca P. (2006). Burkholderia cepacia complex species: Health hazards and biotechnological potential. Trends Microbiol..

[33] Tagele S., Kim S., Lee H., Lee Y. (2019). Potential of Novel Sequence Type of Burkholderia cenocepacia for Biological Control of Root Rot of Maize (*Zea mays* L.) Caused by *Fusarium temperatum*. IJMS.

[34] Chávez-Ramírez B., Kerber-Díaz J.C., Acoltzi-Conde M.C., Ibarra J.A., Vásquez-Murrieta M.-S., Estrada-de los Santos P. (2020). Inhibition of Rhizoctonia solani RhCh-14 and Pythium ultimum PyFr-14 by Paenibacillus polymyxa NMA1017 and Burkholderia cenocepacia CACua-24: A proposal for biocontrol of phytopathogenic fungi. Microbiol. Res..

[35] Tyc O., Song C., Dickschat J.S., Vos M., Garbeva P. (2017). The Ecological Role of Volatile and Soluble Secondary Metabolites Produced by Soil Bacteria. Trends Microbiol..

[36] Elshafie H., Camele I., Racioppi R., Scrano L., Iacobellis N., Bufo S. (2012). In Vitro Antifungal Activity of Burkholderia gladioli pv. agaricicola against Some Phytopathogenic Fungi. IJMS.

[37] Tenorio-Salgado S., Tinoco R., Vazquez-Duhalt R., Caballero-Mellado J., Perez-Rueda E. (2013). Identification of volatile compounds produced by the bacterium Burkholderia tropica that inhibit the growth of fungal pathogens. Bioengineered.

[38] Elkahoui S., Djébali N., Yaich N., Azaiez S., Hammami M., Essid R., Limam F. (2015). Antifungal activity of volatile compounds-producing Pseudomonas P2 strain against Rhizoctonia solani. World J. Microbiol. Biotechnol..

[39] Dolch M.E., Hornuss C., Klocke C., Praun S., Villinger J., Denzer W., Schelling G., Schubert S. (2012). Volatile organic compound analysis by ion molecule reaction mass spectrometry for Gram-positive bacteria differentiation. Eur. J. Clin. Microbiol. Infect. Dis..

[40] Zhang D., Yu S., Yang Y., Zhang J., Zhao D., Pan Y., Fan S., Yang Z., Zhu J. (2020). Antifungal Effects of Volatiles Produced by Bacillus subtilis Against Alternaria solani in Potato. Front. Microbiol..

[41] Tait E., Perry J.D., Stanforth S.P., Dean J.R. (2014). Identification of Volatile Organic Compounds Produced by Bacteria Using HS-SPME-GC–MS. J. Chromatogr. Sci..

[42] Lough F., Perry J.D., Stanforth S.P., Dean J.R. (2017). Detection of exogenous VOCs as a novel in vitro diagnostic technique for the detection of pathogenic bacteria. TrAC Trends Anal. Chem..

[43] Groenhagen U., Baumgartner R., Bailly A., Gardiner A., Eberl L., Schulz S., Weisskopf L. (2013). Production of Bioactive Volatiles by Different Burkholderia ambifaria Strains. J. Chem. Ecol..

[44] Carrión V.J., Cordovez V., Tyc O., Etalo D.W., de Bruijn I., de Jager V.C.L., Medema M.H., Eberl L., Raaijmakers J.M. (2018). Involvement of Burkholderiaceae and sulfurous volatiles in disease-suppressive soils. ISME J..

[45] Kai M., Haustein M., Molina F., Petri A., Scholz B., Piechulla B. (2009). Bacterial volatiles and their action potential. Appl. Microbiol. Biotechnol..

[46] Chambers A.H., Evans S.A., Folta K.M. (2013). Methyl Anthranilate and γ-Decalactone Inhibit Strawberry Pathogen Growth and Achene Germination. J. Agric. Food Chem..

[47] Zhang Z.-Z., Li Y.-B., Qi L., Wan X.-C. (2006). Antifungal Activities of Major Tea Leaf Volatile Constituents toward *Colletorichum camelliae Massea*. J. Agric. Food Chem..

[48] Jantan I.B., Karim Moharam B.A., Santhanam J., Jamal J.A. (2008). Correlation Between Chemical Composition and Antifungal Activity of the Essential Oils of Eight *Cinnamomum*. Species. Pharm. Biol..

[49] Rathna J., Bakkiyaraj D., Pandian S.K. (2016). Anti-biofilm mechanisms of 3,5-di-tert-butylphenol against clinically relevant fungal pathogens. Biofouling.

[50] Jang Y.-W., Jung J.-Y., Lee I.-K., Kang S.-Y., Yun B.-S. (2012). Nonanoic Acid, an Antifungal Compound from *Hibiscus syriacus* Ggoma. Mycobiology.

[51] Moore-Landecker E., Stotzky G. (1973). Morphological Abnormalities of Fungi Induced by Volatile Microbial Metabolites. Mycologia.

[52] Martins S.J., Faria A.F., Pedroso M.P., Cunha M.G., Rocha M.R., Medeiros F.H.V. (2019). Microbial volatiles organic compounds control anthracnose (Colletotrichum lindemuthianum) in common bean (*Phaseolus vulgaris* L.). Biol. Control.

[53] Xing M., Zheng L., Deng Y., Xu D., Xi P., Li M., Kong G., Jiang Z. (2018). Antifungal Activity of Natural Volatile Organic Compounds against Litchi Downy Blight Pathogen *Peronophythora litchii*. Molecules.

[54] Gotor-Vila A., Teixidó N., Di Francesco A., Usall J., Ugolini L., Torres R., Mari M. (2017). Antifungal effect of volatile organic compounds produced by *Bacillus amyloliquefaciens* CPA-8 against fruit pathogen decays of cherry. Food Microbiol..

[55] Mahenthiralingam E., Bischof J., Byrne S.K., Radomski C., Davies J.E., Av-Gay Y., Vandamme P. (2000). DNA-Based Diagnostic Approaches for Identification of Burkholderia cepacia Complex, Burkholderia vietnamiensis, Burkholderia multivorans, Burkholderia stabilis, and Burkholderia cepacia Genomovars I and III. J. Clin. Microbiol..

[56] Jiang L., Jeong J.C., Lee J.-S., Park J.M., Yang J.-W., Lee M.H., Choi S.H., Kim C.Y., Kim D.-H., Kim S.W. (2019). Potential of Pantoea dispersa as an effective biocontrol agent for black rot in sweet potato. Sci. Rep..

[57] Kong W.-L., Li P.-S., Wu X.-Q., Wu T.-Y., Sun X.-R. (2020). Forest Tree Associated Bacterial Diffusible and Volatile Organic Compounds against Various Phytopathogenic Fungi. Microorganisms.

