# Supplementary material for: Characterization of Volatile Organic Compounds Emitted from Endophytic Burkholderia cenocepacia ETR-B22 by SPME-GC-MS and Their Inhibitory Activity against Various Plant Fungal Pathogens
Source: Molecules. 2020 Aug 19;25(17):3765. doi: 10.3390/molecules25173765 (PMC7504634; doi:10.3390/molecules25173765)
Supplement: Supplementary file 1 [file molecules-25-03765-s001.pdf]

molecules

MDPI

Supplementary Materials

Table S1. Volatile organic compounds detected and identified by HS-SMPE GC-MS analysis of *B. cenocepacia* ETR-B22. Retention Time (RT), Chemical formula and GC peak Relative Area (RA), Similarity (S) and Chemical Abstracts Service (CAS) identifier were listed in this table.

Chemical class	Compound	Chemical formula	RT (min)	RA (%)	S (%)	CAS#
hydrocarbons	1,3,5,7-Cyclooctatetraene	C_8H_8	6.14	0.25±0.02	93.2	629-20-9
	Decane, 3,7-dimethyl-	C_8H_{18}	13.33	0.12±0.01	89.0	17312-54-8
	Naphthalene	$C_{10}H_8$	15.74	0.20±0.01	92.7	91-20-3
	Undecane, 2,10-dimethyl-	$C_{13}H_{28}$	22.58	0.21±0.02	87.4	17301-27-8
	Cyclopentane, decyl-	$C_{15}H_{30}$	25.07	0.24±0.02	93.5	541-02-6
	3-Hexadecene, (Z)-	$C_{16}H_{32}$	26.46	0.52±0.07	96.4	34303-81-6
	Eicosane	$C_{20}H_{42}$	27.73	0.13±0.01	92.8	112-95-8
	2,6,10,15-Tetramethylheptadecane	$C_{21}H_{44}$	28.75	0.11±0.02	88.0	54833-48-6
	Pentadecane, 3-methyl-	$C_{16}H_{34}$	28.98	0.18±0.01	92.0	2882-96-4
	Heptadecane	$C_{17}H_{36}$	32.74	0.75±0.11	92.9	629-78-7
	Heneicosane	$C_{21}H_{44}$	43.4	0.11±0.01	86.0	629-94-7
Esters	Methyl benzoate	$C_8H_8O_2$	12.92	0.37±0.08	96.2	93-58-3
	Benzyl acetate	$C_9H_{10}O_2$	15.33	2.14±0.21	97.0	140-11-4
	Methyl salicylate	$C_8H_8O_3$	16.29	0.56±0.08	90.5	119-36-8
	Benzyl propionate	$C_{10}H_{12}O_2$	18.67	0.53±0.06	95.6	122-63-4
	Methyl anthranilate	$C_8H_9NO_2$	21.49	5.17±0.19	97.3	134-20-3
	3-Hexen-1-ol, benzoate, (Z)-	$C_{13}H_{16}O_2$	28.90	0.28±0.05	94.9	25152-85-6
	Benzyl benzoate	$C_{14}H_{12}O_2$	34.32	0.79±0.25	97.2	120-51-4
Ethers	Dimethyl trisulfide	$C_2H_6S_3$	8.91	1.34±0.15	88.5	3658-80-8
	Allyl benzyl ether	$C_{10}H_{12}O$	11.04	0.62±0.08	88.9	14593-43-2

Ketones	2-Pentadecanone	$C_{15}H_{30}O$	32.43	0.18±0.04	88.1	2345-28-0
	2(3H)-Furanone,5-dodecyldihydro	$C_{18}H_{34}O_2$	41.50	0.41±0.05	89.0	502-26-1
Alcohols	2-Isopropyl-5-methyl-1-heptanol	$C_{11}H_{24}O$	20.78	0.19±0.03	87.5	91337-07-4
	trans-Geranylgeraniol	$C_{20}H_{34}O$	37.73	0.09±0.01	86.3	24034-73-9
Organic acids	Nonanoic acid	$C_9H_{18}O_2$	19.04	0.29±0.05	91.2	112-05-0
	cis-Vaccenic acid	$C_{18}H_{34}O_2$	42.21	0.25±0.01	93.4	506-17-2
	Octadecanoic acid	$C_{18}H_{36}O_2$	42.67	0.39±0.06	93.7	57-11-4
Nitrogenous compounds	Oxime-, methoxy-phenyl-	$C_8H_9NO_2$	7.14	3.28±0.27	86.2	NA
	Indole	C_8H_7N	19.89	1.54±0.05	95.6	120-72-9
	Dodecanamide	$C_{12}H_{25}NO$	34.18	0.20±0.02	84.8	1120-16-7
Phenols	3,5-Di-tert-butylphenol	$C_{14}H_{22}O$	26.89	0.62±0.05	92.5	1138-52-9
Aldehydes	Decanal	$C_{10}H_{20}O$	16.90	0.11±0.02	92.6	112-31-2

Note: The number of similarity of VOCs more than 80 are listed in this table. NA indicates that the compound was not matched to the database. Data are presented as means ± standard error.
